# A Multimodal Semantic-Enhanced Attention Network for Fake News Detection

**DOI:** 10.3390/e27070746

**Published:** 2025-07-12

**Authors:** Weijie Chen, Yuzhuo Dang, Xin Zhang

**Affiliations:** National Key Laboratory of Information Systems Engineering, National University of Defense Technology, No. 109 Deya Street, Changsha 410073, China; dangyuzhuo@nudt.edu.cn (Y.D.); zhangxin16@nudt.edu.cn (X.Z.)

**Keywords:** fake news detection, cross-modal fusion, information enhancement, social relations, information theory

## Abstract

The proliferation of social media platforms has triggered an unprecedented increase in multimodal fake news, creating pressing challenges for content authenticity verification. Current fake news detection systems predominantly rely on isolated unimodal analysis (text or image), failing to exploit critical cross-modal correlations or leverage latent social context cues. To bridge this gap, we introduce the SCCN (**S**emantic-enhanced **C**ross-modal **C**o-attention **N**etwork), a novel framework that synergistically combines multimodal features with refined social graph signals. Our approach innovatively combines text, image, and social relation features through a hierarchical fusion framework. First, we extract modality-specific features and enhance semantics by identifying entities in both text and visual data. Second, an improved co-attention mechanism selectively integrates social relations while removing irrelevant connections to reduce noise and exploring latent informative links. Finally, the model is optimized via cross-entropy loss with entropy minimization. Experimental results for benchmark datasets (PHEME and Weibo) show that SCCN consistently outperforms existing approaches, achieving relative accuracy enhancements of 1.7% and 1.6% over the best-performing baseline methods in each dataset.

## 1. Introduction

The exponential expansion of social media platforms has generated an abundance of digital content while simultaneously diversifying information access channels. News has also spread rapidly in multimodal format with the universal benefit of comprehensive content [1], with multimodal news being more eye-catching and persuasive compared to text-only news [2]. However, this simultaneously facilitates the growth of a considerable quantity of fake news, defined as misinformation that is intentionally fabricated or distorted to mislead or deceive news consumers [3]. From an information-theoretic standpoint, the increasing volume and diversity of accessible information directly increase the uncertainty, complexity, and entropy inherent in fake news detection tasks. Consequently, automated fake news detection systems have been developed to identify misinformation on social media platforms, aiming to mitigate its harmful societal effects while ensuring that users receive authentic information.

The essence of the fake news detection problem is regarded as a binary classification problem, where the traditional content-based methods generally determine the authenticity of fake news by analyzing its content through different modalities. Initially, automated fake news detection approaches generally used the textual content to complete semantic feature modeling in order to make judgments [4]. Due to multimedia technology decreasing the accuracy of unimodal detection approaches, researchers then attempted to extract multimodal features for fake news detection [5,6]. Specifically, multimodal learning models [7] have been developed to comprehensively capture both textual and visual features through variational encoders [8] and self-attention mechanisms [9], reducing the cross-modal fusion gap [10]. Moreover, because the background features of the news entity may impact detection accuracy, knowledge graphs have been introduced as additional information to support detection frameworks [11].

The above-mentioned methods simply take into consideration the content without mining the deeper intrinsic structural relations [12]; thus, structure-aware methods have been designed to solve such issues. Specifically, traditional neural network approaches such as RNN and CNN have been utilized to explore the potential structural features. In detail, social relationships are collected for application in the field of fake news detection [13]; these relationships generally consist of the comments following news, the forwarding users, and their additional comments. Moreover, GAT and GCN have proved to be effective at reflecting the structural features of social relations and accurately obtaining representations of node features [14].

**Figure 1:**
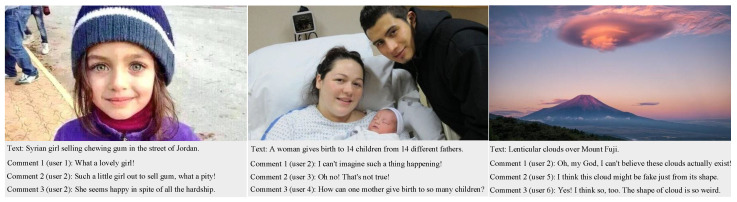
Three pieces of news in the PHEME dataset, including text, images, users, and comments; the top two news stories are in the training set and are labeled as fake, while the last one is in the test set.

Although current approaches have achieved satisfying performance, there remain two main shortcomings. First, simply extracting the feature vectors from text and images fails to capture the entire semantic information, potentially leading to semantic bias in the fusion process of cross-modal features. For example, in the second news story in Figure 1, hardly any clues can be found to prove that it is fake only through the text and image. However, negative opinions arguing that the news may be fake can be read in the comments of different users. Second, the noise in the social relation graphs may lead to bias during information aggregation of nodes by traditional GNNs, resulting in decreased model performance. For example, it is evident that there is a common user (user 2) commenting on all news stories in Figure 1, and their comments on both the first and second news stories take the same position. Obviously, this means there is a high probability that the last news story is fake, even though its text and image provide no evidence to prove that it is. Similarly, there may be some insignificant comments below each news story, such as the first comment on the first news story, the third comment on the second news story, etc. Overall, these comments are irrelevant to the news and could influence the performance of models; thus, they should be removed.

All of the above discussions are centered on the multimodal fake news detection task. Multimodal fake news detection is essentially a simple binary classification task performed to identify whether posts are true or false. Specifically, it aims to map posts to the most likely labels by learning their rich feature inputs, including unimodal features, multimodal features, and social relations. Compared with previous binary classification problems, its uniqueness lies in the use of features from different modalities to improve detection efficiency. For example, text, images, and social relations are all modalities, and the interactive fusion generated among them constitutes multimodal feature fusion, which is a non-negligible factor affecting the classification effect.

In this study, we propose a novel Semantic-enhanced Cross-modal Co-attention Network (SCCN) for multimodal fake news detection. It not only utilizes entity extraction to semantically enhance news but also enables the effective integration of text, images, and social relations. To better capture the structural features, we first model social relations as a graph and make some improvements to GAT. On one hand, we detect latent connections among nodes to enhance the social relation graph while simultaneously reducing noise interference. On the other, we introduce a sign strategy to capture neighbor-to-node correlations and amplify the positive–negative feedback to optimize the node representation. Unlike social relations, we encode text and images directly in addition to enhancing them with entity features. Next, we map their feature vectors to the same semantic space by performing cross-modal alignment operations on different modalities. Finally, self-supervised loss and co-attention mechanisms are introduced to reduce noise and improve the fused feature representation for cross-modal fusion. By learning the social relation graph representation and cross-modal fusion, we can enhance the capability of the model for fake news detection.

The main contributions in this study are summarized as follows:

1. We propose a novel Semantic-enhanced Cross-modal Co-attention Network for multimodal fake news detection, which focuses on utilizing the entity features for semantic enhancement and exploring the social relation graph.
2. We narrow the gap between different modalities by combining the entities and semantics of different modalities. In addition, we introduce self-supervised learning along with co-attention mechanisms for denoising, ultimately enhancing news semantics.
3. We introduce social relations as the structural features and design an improved GAT to process the social relation graph, thereby improving the representations from learning social relation graphs through enhanced neighborhood aggregation.

The rest of this paper is organized as follows: Section 2 reviews the existing approaches for fake news detection and analyzes their shortcomings; Section 3 introduces our proposed SCCN approach; Section 4 provides the experimental details, including the research questions, datasets, baselines, and experimental setup; Section 5 analyzes the results of experiments and discusses the findings of our experiments from different perspectives; finally, Section 6 concludes our current study and outlines prospects for future studies.

## 2. Related Studies

Existing studies on multimodal fake news detection can be categorized into two types: those with semantic-based features and those with structural-based features.

Semantic-based features are expressed not only in text but also in images [15]. This is due to evidence showing that multimodal approaches outperform unimodal approaches in fake news detection [16]. In detail, Wang et al. [2] proposed EANN, an end-to-end model capable of capturing features that are robust to event variations. However, its reliance on annotated data and computationally intensive training processes could limit scalability and training stability. Another end-to-end network is MVAE [1], which utilizes a bimodal variational framework to learn a probabilistic latent variable model. However, its dependence on paired multimodal data and complex architecture could limit its applicability in scenarios with incomplete or single-modality content. In addition, QSAN [17] employs quantum-inspired text analysis and a unique attention system to accurately identify rumors while making its decisions understandable through clear explanations and by highlighting relevant user comments. Due to its complex design and quantum-based computations, this model requires more computing power than traditional detection approaches. Furthermore, Zhou et al. [15] evaluated both the internal relationships within each modality and the cross-modal similarities between text and images for fake news detection. Chen et al. [18] solved the misclassification problem caused by inconsistency across modalities with cross-modal ambiguity learning. In [18], an attention-guided module was proposed to realize more accurate modal alignment and increase the interpretability of modal relationships [19].

However, the aforementioned approaches ignore the rich structural features in news and the plentiful factual information in knowledge graphs. To better explore the structural features in news, researchers have explored ways of representing news content through graph-based structures [20]. For example, EBGCN [21] boosts detection accuracy by dynamically adjusting unreliable edges in rumor propagation graphs; however, its Bayesian framework requires more training data to stabilize probabilistic inferences compared to deterministic models. The GLAN model [13] effectively merges local semantic relationships with global structural patterns through attention mechanisms, achieving superior rumor detection performance. However, its effectiveness can be compromised by noisy data in early propagation phases, and requires precise hyperparameter tuning to maintain optimal performance. Based on Yuan et al. [13], Qian et al. [22] integrated the modalities to capture important information in posts and further utilized a learning principal of knowledge-aware multimodal graphs to adaptively learn features from news stories. MPFN [5] improves fake news identification through the layered integration of text and image data, but requires greater processing power due to its advanced architecture and multi-stage blending components. Finally, MFAN [10] advances rumor detection through its novel integration of multimodal features and attention mechanisms; yet, its effectiveness depends on comprehensive data coverage across all modalities, and requires substantial computational resources for optimal operation.

## 3. Approach

**Figure 2:**
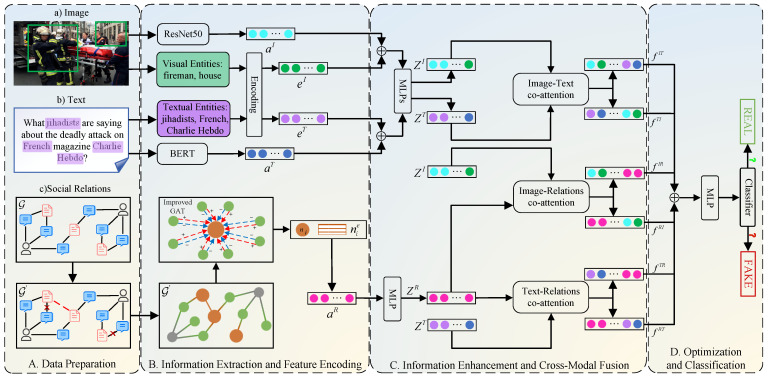
The framework of our proposed SCCN. Specifically, we first extract the features of images, text, and social relations from news stories. We then obtain their feature vectors through feature extraction, where the text and images are the content features and the social relations are the structural features. Meanwhile, we extract entities from images and text and encode them to enhance the content features before performing modal alignment with a self-supervised loss to facilitate subsequent feature fusion. In addition, we obtain the enhanced features between modalities through a co-attention mechanism. Finally, we integrate all the enhanced features of different modalities to obtain a cross-modal fusion feature for fake news detection.

In this section, we detail the proposed SCCN, the framework of which is presented in Figure 2. As demonstrated in Figure 2, SCCN comprises four fundamental components. First, data preparation (Section 3.1) includes the preparation of images, text, and relations along with the problem definition. Second, information extraction and feature encoding (Section 3.2) extracts and encodes the entities from image and text, respectively. In addition, the feature vectors of visual, textual, and social relations are obtained by the corresponding encoders. Third, information enhancement and cross-modal fusion (Section 3.3) enhance the text and image features through entity extraction; meanwhile, an improved GAT is utilized to refine the characteristics of social relations. In addition, we utilize a co-attention mechanism to realize the integration of images, text, and social relations. Finally, optimization and classification (Section 3.4) is mainly used to optimize the cross-modal fusion features and realize fake news detection through classifiers.

Below, we denote the data by mathematical symbols for convenience of description. Specifically, let $D=\left\{X,Y\right\}$ be a set of news on social media containing multimodal features and their labels, which can be denoted as $X=\left\{x_{1},x_{2},\ldots ,x_{n}\right\}$ and $Y=\left\{y_{1},y_{2},\ldots ,y_{n}\right\}$, respectively. For each news $x_{j}=\left\{t_{j},v_{j},u_{j},c_{j}\right\}\in X$, which is a quadruple, $t_{j}$, $v_{j}$, $u_{j}$, and $c_{j}$ represent the text, images, users, and comments, respectively. Moreover, $c_{j}=\left\{c_{j}^{1},c_{j}^{2},\ldots ,c_{j}^{i}\right\}$ denotes the set of comments on news $x_{j}$, where each comment $c_{j}^{i}$ corresponds to a user $u_{j}^{i}$. To better understand the mathematical symbols mentioned above, we take Figure 1 as an example to illustrate the specific meaning of each symbol corresponding to Figure 1. First, the three posts and their labels can be represented as $X=\left\{x_{1},x_{2},x_{3}\right\}$ and $Y=\left\{y_{1},y_{2},y_{3}\right\}$, respectively. Then, we focus on the first post ($x_{1}$), where $t_{1}$ is the text “Syrian girl selling chewing gum in the street of Jordan”, $v_{1}$ is the image in this post, $u_{1}$ contains two users (user 1 and user 2), and $c_{1}$ includes three comments posted by two users. To highlight the connections between news, users, and comments, we build a heterogeneous graph $G=\left\{V,A,E\right\}$, called a social relation graph, where *V* represents the set of nodes, including the above-mentioned three types of nodes. Moreover, $A\in R^{\left|V|*|V\right|}$ is an adjacency matrix which reflects the connections between nodes, i.e., the value is 1 if there is a link between nodes and 0 if there is none, with *E* as the set of edges between nodes.

In this paper, we define fake news detection as a binary classification task. The value of label *y* is 0 or 1, where y=1 represents fake news and y=0 legitimate news. The objective of our study is to predict the label of a given news item *D* through our proposed SCCN model.

### 3.1. Data Preparation

Real-world news stories posted on social media contain rich content and structural features such as images, text, and social relations [23]. Specifically, each news story contains various entities and covers different abundant modalities. The special relationships between different modalities, including images, text, and social relations, provide us with ideas for fake news detection.

In the data preparation section, we extract the data that our proposal requires from the datasets, such as images, text, users, and comments. For the users and their comments on the news, we visualize the relationship between them in a graph, which can be called the social relation graph. To infer hidden links and remove noisy links, it is necessary to process the social relation graph. According to the homogeneity of graph neural networks, we assume that similar nodes are more likely to be associated with each other than dissimilar nodes [24]. Therefore, it is possible to infer the links between nodes with high similarity and remove noisy links between nodes with low similarity by calculating the similarity of features between different nodes.

Specifically, for the embeddings of nodes in G, the vectors of text in news and the vectors of sentences in comments are used as the initial embeddings of news nodes and comment nodes, respectively. Then, we adopt the mean value of all comment embeddings published by one user as this user’s embedding vector. To facilitate the subsequent computation, we construct the node embedding matrix $B\in R^{|V|*d}$, where each row represents the embedding of one node and *d* represents the dimension size of the embeddings.

After that, we use the cosine similarity to calculate the similarity coefficient $\alpha_{ij}$ between nodes $n_{i}$ and $n_{j}$, as follows:

(1) $$\alpha_{ij}=\frac{b_{i}\cdot b_{j}}{∥b_{i}∥∥b_{j}∥},$$

where $b_{i}$ and $b_{j}$ denote the embeddings of nodes $n_{i}$ and $n_{j}$, respectively.

Based on the findings of a previous study [10], we argue that if there is currently no link between nodes, the potential link may exist if the similarity coefficient is above 0.5. Moreover, if there is already a link between nodes, this link can be regarded as noise if the similarity coefficient is below 0.2. This can be formulated as follows:

(2) $$\delta_{ij}=\left\{\begin{array}{cc} -1, & if\;\alpha_{ij}<0.2\\ 0, & if\;0.2\leq \alpha_{ij}\leq 0.5\\ 1, & otherwise \end{array}\right.,$$

where $\delta_{ij}$ is a transition variable that measures the similarity between nodes $n_{i}$ and $n_{j}$.

Next, we improve the original adjacency matrix *A* based on $\delta_{ij}$, i.e., removing the noisy links and adding the potential links, which can be formulated as follows:

(3) $$a_{ij}^{\prime }=\left\{\begin{array}{cc} 0, & if\;\delta_{ij}=-1\;or\;if\;\delta_{ij}=0\;and\;a_{ij}=0\\ 1, & otherwise \end{array}\right.$$

where $a_{ij}$ and $a_{ij}^{\prime }$ are the elements in the initial and improved adjacency matrices *A* and $A^{\prime }$, respectively.

Up to this point, we have enhanced the performance of the model and reduced its training time by removing distracting noise such as malicious comments and bot users from the social relation graph. In this way, we obtain the improved social relation graph $G^{\prime }=\left\{V,A^{\prime },E\right\}$ and input the image, text, and graph $G^{\prime }$ to the next step.

### 3.2. Information Extraction and Feature Encoding

In the information extraction and feature encoding module, we adopt a synchronous processing mode for processing the input data to obtain the required feature vectors, i.e., learning the text and image representations and the social relation graph representation.

#### 3.2.1. Text and Image Representations

First, we extract the corresponding entities from the image and text of each news story using the existing entity linking tool TAGME (https://sobigdata.d4science.org/group/tagme/ (accessed on 27 March 2023)), which can precisely identify the entities and provide their confidence coefficient. Then, we retain the entities with confidence coefficients larger than 0.2 as the final entity extraction results. In addition, a pretrained TransE model [25] attaches them to the Freebase [26] knowledge graph to obtain the background knowledge features $e^{I}$ and $e^{T}$ as the entity embeddings.

Subsequently, we extract the feature vectors from the text and image using the respective encoders. Specifically, we utilize the pretrained BERT [27] and ResNet-50 [28] as the encoders for text and image, respectively. However, the length of the text generally varies for different news stories. Therefore, to facilitate subsequent operations, we set the text of different news to be of the same length, i.e., *L*, through padding or truncating, which can be denoted as follows:

(4) $$T^{i}\in R^{L*d}=\left\{t_{1}^{i},t_{2}^{i},\ldots ,t_{L}^{i}\right\}$$

where *d* is the dimension of the word embeddings and $T^{i}$ is the text embedding of the *i*-th news item, which consists of *L* word embeddings.

Next, this embedding sequence is fed into Bi-LSTM [29] to obtain the text feature vector $a^{T}$, as follows:

(5) $$a^{T}=W_{T}\left(Bi-LSTM\left(T^{i}\right)\right)+b_{T}$$

where $W_{T}$ is the learnable weight matrix and $b_{T}$ is the bias vector.

For the image, we extract the output of the second-last layer of ResNet-50 [28] and subsequently feed it through a fully connected layer to generate a feature vector $a^{I}$ with the same dimensions as the text feature $a^{T}$. This can be formulated as

(6) $$a^{I}=sigmoid\left(W_{I}*R_{I}\right),$$

where $R_{I}$ is the output of the second-last layer of ResNet-50 and $W_{I}$ is the weight matrix of the fully connected layer.

#### 3.2.2. Social Relation Graph Representation

**Figure 3:**
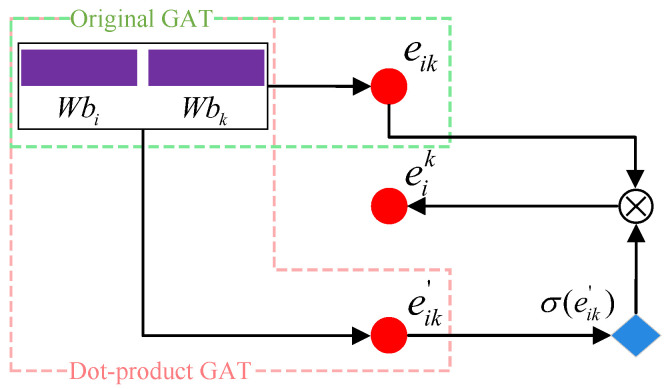
Pipeline of the improved GAT, where we combine the green and red box parts to compose a new mixed form. The red circles represent attention that is not normalized, while the blue diamond is the transition node.

Unlike the above two types of representations, the social relation graph contains abundant structural features. Inspired by Velickovic et al. [30], Graph Attention (GAT) networks can capture the graph structure features for graph processing. However, traditional GAT suffers from poor interpretability and inconsistent performance across datasets when the processed graphs are complicated [24]. Thus, we propose an improved GAT to capture the correlations of the neighbor nodes for better graph feature representation, the pipeline of which is shown in Figure 3.

Attention Weight

Specifically, for node $n_{i}$ and its set of neighbor nodes $N_{i}=\left\{n_{i}^{1},n_{i}^{2},\ldots ,n_{i}^{k}\right\}$ in graph $G^{\prime }$, we first calculate the attention weights $E_{i}=\left\{\varepsilon_{i}^{1},\varepsilon_{i}^{2},\ldots ,\varepsilon_{i}^{k}\right\}$, where the element $\varepsilon_{i}^{k}\in E$ denotes the attention weight between $n_{i}$ and $n_{i}^{k}$. In detail, it can be seen in Figure 3 that we integrate two common attention mechanisms of the traditional GAT, i.e., the single-layer neural network and the dot product [30]. This can be formulated as

(7) $$\left\{\begin{array}{c} e_{i}^{k}={\tilde{a}}^{⊤}\left[Wb_{i}∥Wb_{k}\right]\cdot sigmoid\left({\left[Wb_{i}\right]}^{⊤}\cdot Wb_{k}\right)\\ \varepsilon_{i}^{k}=LeakyReLU\left(e_{i}^{k}\right) \end{array},\right.$$

where ‖ is the concatenation operation, $\tilde{a}$ is a parameter in the single-layer neural network, *W* is a learnable weight matrix, and $b_{i}$ and $b_{k}$ are the embeddings of node $n_{i}$ and its neighbor node $n_{i}^{k}\in N_{i}$, respectively.

Attention Coefficient

Next, to facilitate the following calculation, we employ the softmax function to normalize the obtained attention weight set $E_{i}$. In addition, we note that some of the attention weights become very small after normalization, which means that these neighbor nodes have very little influence on node $n_{i}$. Such situations generally occur when the attention weights are negative. In fact, the attention weights objectively reflect the potential influence of the neighbor nodes on node $n_{i}$, which include both positive and negative influences, and these influences’ reflection in the values of the attention weights is correspondingly positive or negative. However, the direct application of the softmax function ignores the negative influence, which we also need to focus on.

For instance, given a specific node $n_{p}$, we obtain its attention weights as $E_{p}=\left\{0.7,0.2,0.1,-0.2,-0.8\right\}$; the subsequently obtained attention coefficients after normalization are $\Phi_{p}=\left\{0.36,0.22,0.20,0.14,0.08\right\}$. It can be observed that the neighbor node with attention weight $\varepsilon_{p}^{5}=-0.8$ has a normalized attention coefficient $\phi_{p}^{5}=0.08$, which indicates that its contribution to the output is almost negligible. This large negative influence implies that the embeddings of these two nodes are in opposite directions, which may be beneficial for fake news detection. This situation may be an instance of “cheating” or “camouflage” [14]. In certain cases, there may be paid-for real comments on fake news stories or malicious comments added to discredit real news [14].

As a result, inspired by [17], we introduce a sign mechanism to correctly handle the positive and negative relations between nodes. In detail, we obtain ${\tilde{E}}_{i}$ after taking the opposite number of attention weights $E_{i}$ for node $n_{i}$, i.e., ${\tilde{E}}_{i}=-E_{i}=\left\{{\tilde{\varepsilon }}_{i}^{1},{\tilde{\varepsilon }}_{i}^{2},\ldots ,{\tilde{\varepsilon }}_{i}^{k}\right\}$. Afterwards, normalization is implemented with the softmax function for the two attention weights $E_{i}$ and ${\tilde{E}}_{i}$ to obtain the corresponding attention coefficients. This can be formulated as follows:

(8) $$\left\{\begin{array}{c} \phi_{i}^{j}=softmax_{j}\left(\varepsilon_{i}^{j}\right)=\frac{exp\left(LeakyReLU\left(\varepsilon_{i}^{j}\right)\right)}{\Sigma_{k\in |N_{i}|}exp\left(LeakyReLU\left(\varepsilon_{i}^{k}\right)\right)}\\ \phi_{i}^{j^{\prime }}=softmax_{j}\left({\tilde{\varepsilon }}_{i}^{j}\right)=\frac{exp\left(LeakyReLU\left({\tilde{\varepsilon }}_{i}^{j}\right)\right)}{\Sigma_{k\in |N_{i}|}exp\left(LeakyReLU\left({\tilde{\varepsilon }}_{i}^{k}\right)\right)} \end{array}\right.$$

where $\phi_{i}^{j}\in \Phi_{i}$ and ${\tilde{\phi }}_{i}^{j}\in {\Phi_{i}}^{\prime }$ are the two attention coefficients.

Graph Feature

To completely capture the interactions between nodes, we obtain the weighted aggregation of the neighbor nodes of $n_{i}$ with $\Phi_{i}$ and ${\Phi_{i}}^{\prime }$, respectively. Subsequently, the above vectors are concatenated and fed into a fully connected layer to obtain the final vector representation of node $n_{i}$. Note that we utilize multihead attention in order to adapt to the complex graph structure in this process. This allows the model to fully take into account the correlation and importance between different nodes, resulting in improved expressive ability. The multihead attention can be formulated as follows:

(9) $$b_{i}^{\prime }\left(K\right)=\overset{K}{\underset{k=1}{∥}}\sigma \left(W^{k}*\left(\Phi_{i}^{k}*B_{i}∥-{\Phi_{i}^{k}}^{\prime }*B_{i}\right)\right)$$

where *K* is the number of heads, σ is the activation function, $W^{k}$ is the weight matrix of the fully connected layer of the *k*-th head, and $B_{i}$ is the embedding matrix of the neighbor nodes.

To this point, the final embeddings of all nodes are calculated by Equation (9) and denoted as the node embedding matrix $B^{\prime }$. Finally, the multihead attention mechanism [31] is employed to acquire the features of the social relation graph as follows:

(10) $$G=\overset{K}{\underset{k=1}{∥}}\sigma \left(B^{\prime }\right)$$

where *K* is the number of heads and the *i*-th column of *G* denotes the graph feature of the *i*-th news item.

### 3.3. Information Enhancement and Cross-Modal Fusion

In this section, we perform information enhancement and cross-modal fusion using the feature vectors obtained from Section 3.2 for text, image, and the social relation graph. For the feature vectors of the text and images, we use their entity embeddings to implement self-information enhancement. Specifically, taking an image as an example, we concatenate $a^{I}$ and $e^{I}$ before feeding them into a multilayer perceptron to obtain the information-enhanced feature vector $Z^{I}$. Moreover, the information-enhanced feature vector $Z^{T}$ of the text can be calculated in the same way. For the social relation graph, we acquire the feature vector $Z^{R}$ with the same dimension as $Z^{I}$ by feeding *G* into a multilayer perceptron [31]. This can be formulated as

(11) $$\left\{\begin{array}{c} Z^{I}=\sigma \left(W_{I}^{\prime }\left(a^{I}∥e^{I}\right)+b_{I}^{\prime }\right)\\ Z^{T}=\sigma \left(W_{T}^{\prime }\left(a^{T}∥e^{T}\right)+b_{T}^{\prime }\right)\\ Z^{R}=\sigma \left(W_{R}^{\prime }G+b_{R}^{\prime }\right) \end{array},\right.$$

where σ is the activation function, $W_{I}^{\prime }$, $W_{T}^{\prime }$, and $W_{R}^{\prime }$ are the learnable weights matrix, and $b_{I}^{\prime }$, $b_{T}^{\prime }$, and $b_{R}^{\prime }$ are the bias vectors.

It cannot be ignored that performing the cross-modal fusion operations inevitably causes intrinsic loss of information between modalities, which should be intrinsic to the representation of different modalities in the original news story. This leads to the features extracted from disparate modalities potentially exhibiting substantial semantic gaps. To address this issue, a novel cross-modal alignment with self-supervised loss is introduced to refine the feature representations. For example, we map the produced feature vectors $Z^{I}$ and $Z^{T}$ to the same semantic space as, follows:

(12) $$\left\{\begin{array}{c} \hat{Z^{I}}=\hat{W_{I}}Z^{I}\\ \hat{Z^{T}}=\hat{W_{T}}Z^{T} \end{array}\right.$$

where $\hat{W_{I}}$ and $\hat{W_{T}}$ are the learnable parameters. After this, we adopt the MSE loss to narrow the distance between $\hat{Z^{I}}$ and $\hat{Z^{T}}$:

(13) $$L_{align}^{TI}=\overset{n}{\underset{i=1}{\sum }}\frac{{\left(\hat{Z^{I}}-\hat{Z^{T}}\right)}^{2}}{n}$$

where *n* is the total number of news stories.

Similarly, we can map $Z^{T}$ and $Z^{R}$ along with $Z^{I}$ and $Z^{R}$ to the same semantic space and calculate their MSE loss as $L_{align}^{TR}$ and $L_{align}^{IR}$, respectively. Then, we add the above three losses to obtain the final loss of the cross-modal alignment, as follows:

(14) $$L_{align}=L_{align}^{TI}+L_{align}^{TR}+L_{align}^{IR}.$$

Up to this point, we have obtained the cross-modal aligned graph feature vectors of images, text, and social relations as $\tilde{Z^{I}}$, $\tilde{Z^{T}}$, and $\tilde{Z^{R}}$, respectively. In addition, considering that there are three feature vectors from different modalities, we need to integrate their embeddings in order to improve the credibility before detection. Inspired by Lu et al. [32], we adopt a cross-modal fusion approach with a co-attention mechanism.

Specifically, we adopt the co-attention mechanism between every two modalities to obtain the cross-modal features containing the key information between the two corresponding modalities. Taking the fusion of text and images as an example, we use $Q_{T}=\tilde{Z^{T}}W_{T}^{Q}$, $K_{I}=\tilde{Z^{I}}W_{I}^{K}$, and $V_{I}=\tilde{Z^{I}}W_{I}^{V}$ to calculate the query matrix, key matrix, and value matrix, respectively. In this case, $W_{T}^{Q}$, $W_{I}^{K}$, and $W_{I}^{V}\in R^{d*\frac{d}{K}}$ are the linear transformation matrices, where *K* is the number of heads and *d* is the dimension of the feature vectors.

Then, we can produce the cross-modal feature $f^{TI}$ between the text and images, calculated as follows:

(15) $$f^{TI}=\left[\overset{K}{\underset{k=1}{∥}}softmax\left(\frac{Q_{T}{K_{I}}^{⊤}}{\sqrt{d}}\right)V_{I}\right]W_{TI}$$

where $W_{TI}$ represents the linear transformation matrix.

However, it is evident that $f^{TI}$ is actually the visual feature enhanced by a textual feature, which does not fully reflect the characteristics and relations between the two modalities. Therefore, we exchange the representation of text and image in Equation (15) to obtain another cross-modal feature $f^{IT}$, which is the textual feature enhanced by the visual feature. For the other two groups of modalities, we obtain the mutually reinforced cross-modal features in the same way. For convenience of distinction, we denote these as $f^{TR}$, $f^{RT}$, $f^{IR}$, and $f^{RI}$, respectively. Finally, we concatenate them as the final multimodal fusion feature, as follows:

(16) $$Z=concat\left(f^{TI},f^{IT},f^{TR},f^{RT},f^{IR},f^{RI}\right).$$

### 3.4. Optimization and Classification

In the optimization and classification module, we feed the final multimodal fusion feature *Z* into a fully connected layer to predict the labels of news stories, as follows:

(17) $$\hat{y}=softmax\left(MLP\left(Z\right)\right),$$

where $\hat{y}$ denotes the predicted scores.

In our model, the entropy can be used to measure the dispersion and uncertainty of the experimental results. In the proposed framework, high entropy reflects significant divergence between detection results and ground-truth labels, whereas low entropy indicates stable convergence. Information theory offers quantitative frameworks for three critical aspects of probabilistic systems: entropy for uncertainty assessment, KL divergence for distributional differences, and mutual information for dependency measurement. Inspired by the theoretical understanding of entropy in information theory, we adopt the cross-entropy loss function as the loss function for this binary classification problem. The optimization strategy of SCCN explicitly targets entropy minimization through cross-modal feature learning, as follows:

(18) $$L_{cls}=-ylog\left(\hat{y}\right)-\left(1-y\right)log\left(1-\hat{y}\right)$$

where *y* indicates the ground truth of the fake news detection label.

Considering that cross-modal alignment is conducted to narrow the semantic gaps between modalities, the generated loss may contribute less to the final classification. Thus, we introduce two parameters, $\lambda_{a}$ and $\lambda_{b}$, to respectively regulate $L_{cls}$ and $L_{align}$ in the total loss function. We then combine $L_{cls}$ and $L_{align}$ to compute the final loss function of SCCN, as follows:

(19) $$L_{total}=\lambda_{a}L_{cls}+\lambda_{b}L_{align}.$$

However, we find that the text of some news items does not strictly follow the grammar rules in practice, which may reduce the efficiency of our model to some extent. To resolve this issue, we add an adversarial perturbation mechanism, i.e., the Projected Gradient Descent (PGD) [33], to enhance the robustness of our model when extracting the text embeddings. The effectiveness of PGD has been proven by Madry et al. [33]. In detail, we compute the gradients of the text features in training and add perturbations to the text features. We then recalculate its gradient and repeat the above process *T* times. Finally, all the adversarial perturbations are accumulated into the original gradient and the parameters are updated.

More details of the PGD are provided in Algorithm 1. Specifically, in this algorithm, sign(g) is a function that returns the sign of the gradient *g*, indicating the direction in which the input should be perturbed in order to maximize the loss. Moreover, clip and project are functions which respectively ensure that the perturbations are within acceptable bounds and that the perturbed inputs are projected back into the valid data space.
**Algorithm 1** Procedure of PGD**Require:** 
The original dataset $D={\left\{x_{i},y_{i}\right\}}_{i=1}^{N}$. The initialized model parameter θ. Set the epoch number *T*, the step size β and the perturbation limit δ.**Ensure:** 
The model parameter $\theta^{*}$.1:Initialize θ randomly.2:**for** each epoch t=1 to *T* **do**3:    **for** each batch $\left(x_{batch},y_{batch}\right)$ in *D* **do**4:          Calculate the gradient of the loss function:5:          $g={▽}_{x}J\left(\theta ,x_{batch},y_{batch}\right)$.6:          Compute the perturbation in the direction of the gradient:7:          $p=\beta *sign\left(g\right)$8:          Apply the perturbation to the data, ensuring it does not exceed the limit δ:9:          $x_{adv}=x_{batch}+p$   $x_{adv}=clip\left(x_{adv},x_{batch}-\delta ,x_{batch}+\delta \right)$10:        Project the perturbed data back into the valid data space:11:        $x_{adv}=project\left(x_{adv},\left[0,1\right]\right)$12:        Update the model parameter θ:13:        $g_{\theta }={▽}_{\theta }J\left(\theta ,x_{adv},y_{batch}\right)$    $\theta =\theta -\beta *g_{\theta }$14:    **end for**15:**end for**16:**return** the updated $\theta^{*}$.

## 4. Experiments

In this section, we first introduce the datasets in the social media field used to evaluate performance of SCCN in Section 4.1. Next, the discussed baselines are listed in Section 4.2. Finally, we describe the implementation details of our experiments in Section 4.3.

### 4.1. Datasets

The performance of the proposed SCCN and the baselines are evaluated using two widely used datasets: the PHEME [34] and Weibo [35].

***The PHEME Dataset*** (https://figshare.com/articles/PHEME_dataset_of_rumours_and_non-rumours/4010619 (accessed on 24 October 2016)) is a collection of posts on Twitter about multiple breaking news stories and their related information. Similarly, ***the Weibo Dataset*** (https://drive.usercontent.google.com/download?id=14VQ7EWPiFeGzxp3XC2DeEHi-BEisDINn&export=download (accessed on 22 May 2021)) is a Chinese dataset that collects a large quantity of posts from the most widely used social media platforms within China. Both of the above datasets contain rich information derived from news stories, including text, images, users, and comments. In our model, we mainly focus on text, images, and social relation networks to detect fake news. Thus, we perform data cleaning on the raw datasets. We delete those news stories that have only unimodal information such as text or images, as our model is focused on multimodal conditions. Moreover, we make sure that each news story contains at least one piece of information that we need in our model, including text, images, and social relations. The processed statistics relating to the PHEME and Weibo datasets are shown in Table 1.

### 4.2. Baselines

We validate the effectiveness of our proposed SCCN by comparing it with the following competitive baseline models:

- **EANN** [2]: EANN utilizes a cross-modal feature extractor and a fake posts detector to support fake news detection, which can derive event-invariant features that make it easier to detect newly emerging events.
- **MVAE** [1]: MVAE uses a bimodal variational auto-encoder to model images and text in order to achieve classification.
- **QSAN** [17]: QSAN incorporates quantum-driven text encoding along with a signing mechanism within its framework, which can utilize conflicting information to provide clues for detection. In addition, this method is interpretable.
- **SAFE** [15]: SAFE is a fake news detection approach that emphasizes the similarity between textual and visual content more than other methods.
- **EBGCN** [21]: EBGCN identifies unreliable relationships existing in rumors and enables the detection of fake news by training an edge consistency framework.
- **GLAN** [13]: GLAN is a global–local network that captures structural information for fake news detection by jointly coding global and local information.
- **MPFN** [5]: MPFN is able to recognize the level of information represented in the different modalities and use this to build a strong hybrid modality.
- **KMAGCN** [22]: KMAGCN is an adaptive graph convolutional network that converts posts into graphs to capture discontinuous semantic relations.
- **MFAN** [10]: MFAN introduces the element of comments in posts while considering the complement and alignment between different modalities for better integration.

### 4.3. Implementation Details

Following previous studies Zheng et al. [10], we divide the PHEME and the Weibo datasets into a training set, validation set, and test set in a ratio of 7:1:2, respectively. We then sequentially initialize the word embeddings in our model with vectors of dimension 300, i.e., d=300. Those words that are not in the pretrained word vectors are initialized from a uniform distribution. The number of heads *K* is set to 8 for all the multi-head attention mechanisms involved in this paper. The values of $\lambda_{a}$ and $\lambda_{b}$ in Equation (19) are 2.15 and 1.55, according to previous experience. In our experiments, the learning rate during data training is set to 0.002 and the max length L=50. The convolutional kernel sizes are configured to (3, 4, 5), with 100 kernels allocated for each size. The final result of the model is determined by taking the average of the results obtained from five successive runs. Finally, we measure the performance of our model using the common indicators of accuracy, precision, recall, and F1 score.

## 5. Results and Discussion

In Section 5.1, we evaluate the overall performance of our proposed SCCN and the baselines. Following that, we conduct an ablation study to investigate the impact of each component within our proposal in Section 5.2. We then perform a quantitative analysis to visually evaluate the performance of the SCCN and its variants in Section 5.3 based on an ablation study, as well as comparing it to several baselines for further verification of its validity. Next, we perform a convergence analysis to verify the overfitting risk of the proposed SCCN in Section 5.4. In Section 5.5, we analyze the influence of hyperparameters on our model from different angles. Finally, to further understand our SCCN model, we conduct case studies in Section 5.6.

### 5.1. Overall Performance

First of all, we evaluate the fake news detection performance of the SCCN as well as the baselines in Table 2.

For the discussed baselines, we find that the performance of the models that consider social relations is much better than that of the other methods. Note that the baselines listed here are all multimodal fake news detection methods. In detail, the KMAGCN model achieves up to 3.1% and 1.1% performance improvement over the MPFN model on the PHEME and Weibo datasets, respectively. This illustrates the importance of capturing structural information from social relations for fake news detection.

Next, we zoom in on the comparisons of our SCCN against the baselines. Generally, it is evident that SCCN exhibits superior performance in comparison to all the baselines on both the PHEME and Weibo datasets. For instance, SCCN achieves respective performance improvements of up to 1.7% and 1.6% over the best baseline method on the PHEME and Weibo datasets. We consider that the advantage of SCCN is due to its ability to combine the entities and semantics of different modalities along with its optimized representation of social relation graphs.

Because the PHEME dataset is sourced from Twitter, most of the posts are related to specific things and the correlations between them are minor; as a result, this dataset is more likely to result in overfitting. Nevertheless, SCCN performs better on both datasets. This advantage can be explained by the fact that SCCN leverages a co-attention mechanism to enhance text and image representations with entity features. Thus, it can refine feature vectors, which is more conducive to subsequent fusion. In addition, we introduce social relation graphs consisting of news, users, and their comments in order to extract the potential structure features from news items, allowing SCCN to further complete the feature representation.

To further explore the practical deployability of SCCN, we use the three metrics of time, inference latency, and memory. Table 3 shows the time, inference latency, and memory of SCCN on the PHEME and Weibo datasets, respectively. Based on these results, all three capability metrics for our model are within a reasonable range of intervals, showing good potential for practical deployment.

### 5.2. Ablation Study

Next, we perform an ablation study to gain deeper insights into each component of our proposed SCCN and the different modal embeddings contained within it.

#### 5.2.1. Effect of Modules

To assess the impacts of key components of SCCN on detection performance, we construct the following model variants: (1) SCCN w/o DPS: Removes the processing of all raw data in the data preparation section and replaces it with the direct utilization of raw data as input. (2) SCCN w/o SRG: Eliminates the social relations component, considering only the text and image modalities for final fake news detection. (3) SCCN w/o IEM: Replaces the output of the information enhancement modules with the original embedding vectors for the subsequent cross-modal fusion operation. (4) SCCN w/o CMF: Replaces the output of the cross-modal fusion module with the sum of the feature vectors for text, images, and social relations.

Table 4 shows the results of the ablation experiments. We use the accuracy and F1 score indicators, as these most clearly reflect the performance of the different models. Overall, the different SCCN variants all underperform the original model in terms of accuracy and F1 score, suggesting that each module in our model makes a significant contribution to the final performance. Specifically, we summarize the following points:

- Comparing the four variants of SCCN, we find that the SCCN w/o DPS, SCCN w/o SRG, and SCCN w/o IEM variants all show obvious performance drops, indicating that introducing data processing, social relations, and information enhancement strengthens the performance of our model. From an information-theoretic perspective, greater performance degradation indicates higher uncertainty and entropy in the variants, demonstrating enhanced effectiveness of our model architecture when incorporating the proposed modules. Moreover, the results of the ablation study indicate that combining text, images, and social relations can facilitate cross-modal feature fusion in a way that is crucial for fake news detection.
- The performance decrease for the SCCN w/o CMF variant proves that the cross-modal fusion module implemented with the co-attention mechanism helps to improve the performance of our model. Furthermore, all variants show similar performances on both the PHEME and Weibo datasets, while the complete SCCN model performs better on Weibo than PHEME dataset, demonstrating that these modules play a greater role in the Weibo dataset.

#### 5.2.2. Effects of Different Types of Modal Embeddings

**Figure 4:**
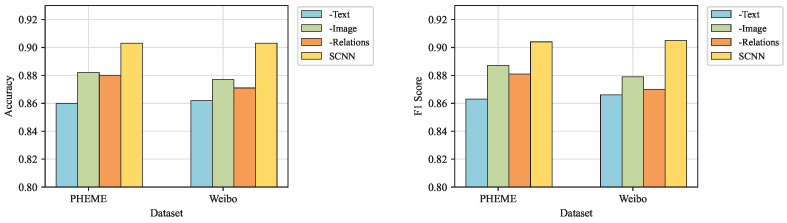
Comparison of accuracy and F1 score when removing different types of modal embeddings.

To more deeply investigate the effects of different modalities and fusion strategies on detection efficacy, the next experiment removes specific modalities from the model. In Figure 4, **-Text** indicates the removal of the text modality, **-Image** indicates the removal of the image modality, and **-Relations** indicates the removal of the social relations modality.

As shown in Figure 4, the full SCCN model outperforms all other variants with different types of modal embeddings. Moreover, in comparing the three variants it can be seen that the detection effect is greatly reduced by removing the text modality, which means that the textual content is more important for fake news detection. We consider the cause of this phenomenon to be the fact that the textual description usually reflects the essential features of a post more accurately, while visual features and social relations may be contaminated by less critical or even irrelevant information [36]. Similarly, **-Image** outperforms **-Relations**, which is due to the social relation graph containing comment nodes. Moreover, comment nodes contain information about users’ comments, which is also represented as textual content. Thus, the text modality assumes a more prominent role in detection.

### 5.3. Quantitative Analysis

**Figure 5:**
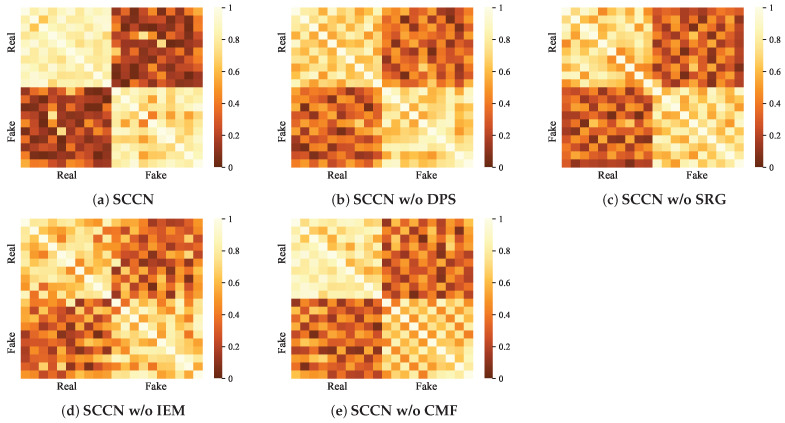
Visualization of the results of SCCN and its variants on the PHEME dataset. The values in the boxes indicate the correlation with the news, which is represented by the correlation of the news prediction labels.

**Figure 6:**
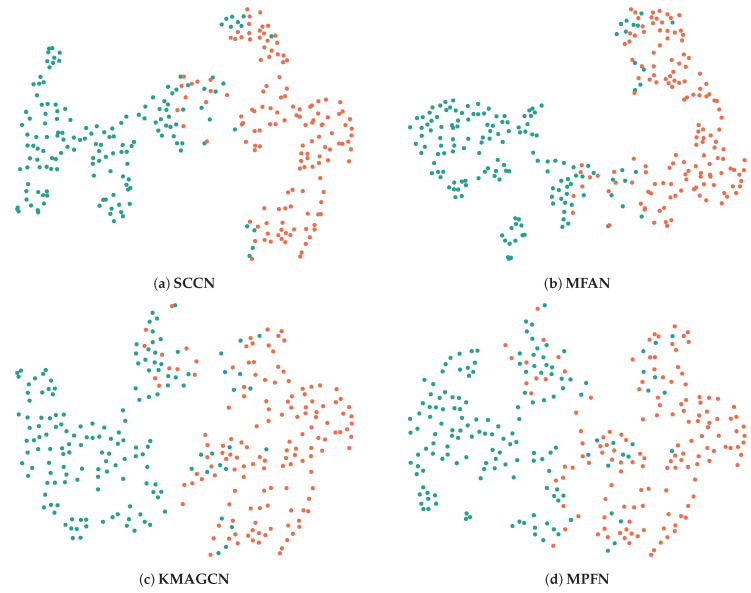
Visualization results of SCCN and several baselines on the Weibo dataset. Dots of the same color belong to the same label.

The ablation experiments allow us to obtain a basic view of the respective impacts of the different modules on the whole model. To more intuitively show the contribution of each module in our model, we visualize the SCCN and its variants on the PHEME dataset. In detail, we adopt heat maps to realize data visualization for the PHEME dataset. Considering that most of the posts in the PHEME dataset are related to some specific events, we randomly select twenty news items from the dataset, including ten pieces of real news and ten pieces of fake news, then input them into the SCCN model and its variants to obtain Figure 5. Meanwhile, we use t-SNE visualization to perform an in-depth analysis of the features learned by SCCN and several baselines, with the results shown in Figure 6.

Figure 5 compares the fake news detection capabilities of our model and its variants using the PHEME dataset. We observe that SCCN provides clear boundaries between real and fake news while showing similarities between the classes. However, the rest of the methods tend to blur the boundaries between real news and fake news, meaning that their performance is poorer and that they are less likely to recognize fake news. The visualization results here are similar to those of the ablation experiments in Section 5.2, with both showing that the performance of the SCCN w/o SRG and SCCN w/o IEM variants is inferior to that of SCCN w/o DPS and SCCN w/o CMF. This suggests that our visualization experiments are consistent with the performance results of the ablation experiments, providing a certain degree of credibility. Social relations are not considered by the SCCN w/o SRG variant, which only integrates text and image features are; this may result in some critical information hidden in the comments being missed. Moreover, the lack of structure information can lead to difficulty in excavating the correlations between news stories to some extent, resulting in reduced model performance. Compared to the SCCN, the SCCN w/o IEM variant not only fails to utilize entity features for the information enhancement of text and image modalities but also struggles to process the information in the social relation graphs without the improved GAT. This can lead to the omission of important information when processing modal information; in other words, it can amplify the noise generated by non-essential information, which can seriously affect model performance. In general, each module of our proposed model plays a favorable role in fake news detection in both the ablation experiments and the quantitative analysis.

Unlike the above format, we next use the t-SNE visualization method to visualize the test set data of the Weibo dataset, as shown in Figure 6. We choose the three most representative baselines for comparison with our model, of which only MPFN does not utilize social relations. Specifically, the boundary between real news and fake news is clear and obviously noticeable with SCCN, indicating that its classification effect is the best among the different models. Similarly, the boundaries obtained with MPFN seem rather indistinct compared to the first three models. It is hypothesized that this phenomenon may be attributable to underutilization of certain social relationships within the dataset, such as users and comments. From an information-theoretic perspective, when feature representations from distinct categories appear in close proximity within the visualization space, this suggests reduced inter-class informational divergence and increased mutual information between categories, both of which represent undesirable characteristics for effective classification. Additionally, the visualization results show general consistency with those in Table 2, demonstrating the rigor of our experiments.

### 5.4. Convergence Analysis

**Figure 7:**
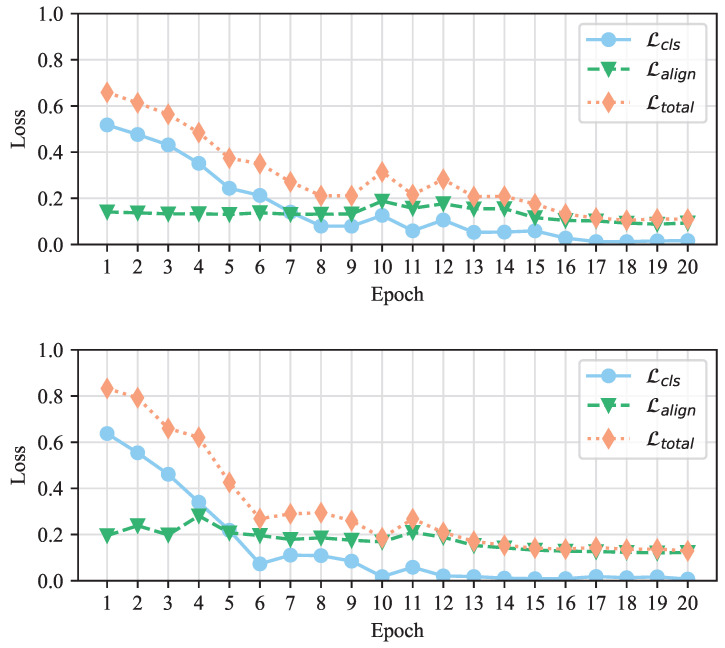
Average training loss on the PHEME and Weibo datasets.

To assess the contributions of $L_{cls}$ and $L_{align}$ during training and verify the convergence of our SCCN model, we generate average loss curves for SCCN in Figure 7. It is evident that the total loss results on both the PHEME and Weibo datasets show a decreasing trend before eventually stabilizing. This is an effective indication that our model has good stability and robustness. In addition, we provide the values of $L_{cls}$ and $L_{align}$ at each epoch, finding that the initial value of $L_{cls}$ is higher and the overall downward trend is more obvious than that of $L_{align}$. We believe that the reason for this phenomenon is that there are too many modules in the model. This causes large classification loss in the whole process, although it still plays an important role in training.

### 5.5. Parameter Analysis

Our proposed SCCN method adopts fixed lengths for the text and convolution kernel sizes; thus, selecting these hyperparameters may have a significant effect on its performance. In the next experiments, we examine the parameter sensitivity in order to analyze the influence of different hyperparameters in our model. According to the principle of control variables, we minimize the influence of other irrelevant parameters. We then demonstrate the feasibility of the parameter settings by performing parameter sensitivity experiments on the text length and the size of the convolution kernel.

#### 5.5.1. Length of Text

Based on the objective fact of text length in the datasets, we set the length of text $L=\left\{20,30,40,50,60\right\}$ and record the accuracy and F1 score. Table 5 demonstrates the performance of the model with different text lengths. It can be seen that the text length has an effect on our model to some extent. In detail, the performance of SCCN decreases if the text length is too short or too long. A possible reason for this trend is that when the text length is too short, it cannot provide sufficient information for SCCN to accurately distinguish fake news. Moreover, although longer text lengths can provide enough information, they introduce noise which may affect the performance of the model.

#### 5.5.2. Size of the Convolution Kernel

**Figure 8:**
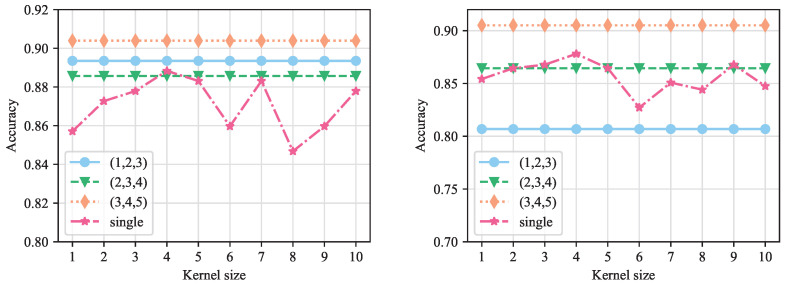
Accuracy of different convolutional kernel sizes on the PHEME and Weibo databases. Here, “single” indicates a single fixed convolutional kernel size.

Figure 8 shows the accuracy comparison of different convolution kernel sizes on the PHEME and Weibo, where the x-axis indicates the kernel size. Experiments on both datasets indicate that when the kernel size is set to a single fixed value, the performance increases as the kernel size increases, peaking around sizes of 3 and 4. In addition, we conduct comparative experiments utilizing various convolutional kernel configurations. The results demonstrate that a hybrid architecture incorporating kernel sizes of 3, 4, and 5 yields optimal detection accuracy; consequently this configuration is implemented in the full SCCN model. The effectiveness of combining multiple convolution kernel sizes can be understood based on the fact that different kernel sizes can help the model to capture distinctive information from multiple perspectives, which is conducive to promoting the exploration of potential key information.

### 5.6. Case Studies

To further illustrate the importance of social relations and entity extraction in the proposed SCCN, we provide some examples of fake news identified by our model to demonstrate its effectiveness.

**Figure 9:**
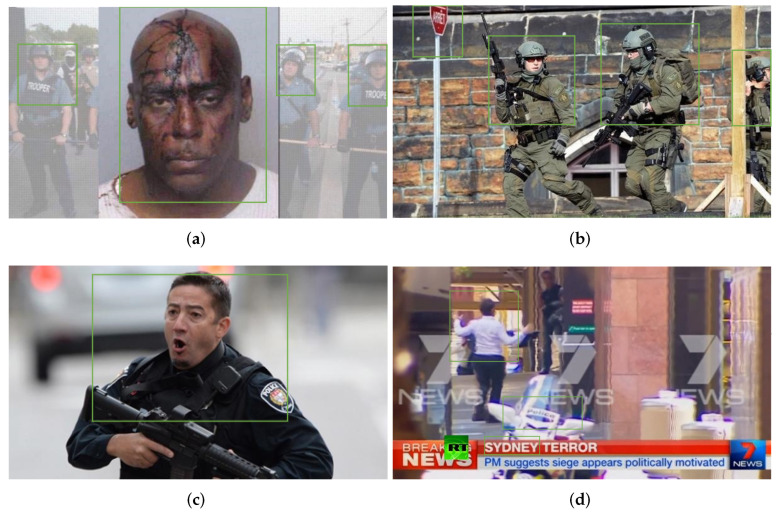
Images and text of example fake news stories detected by SCCN: (**a**) “This is what **#Ferguson** PD did to **#HenryDavis** in 2009 when they mistook him for a man with an outstanding warrant”; (**b**) “According to reports, one **gunman** is dead in **#Ottawa** Parliament shooting”; (**c**) “**Ottawa** shooting: **Soldier** dies of injuries, **gunman** shot dead”; (**d**) “DRAMATIC VIDEO: **#Sydney** siege hostages run from **#Lindt Cafe**, more remain trapped”.

Figure 9 shows an example of fake news detected by our proposed SCCN model on the PHEME test set. In Figure 9a,b, the attached image and text examples appear normal without any anomalies, and there is no significant difference in the content that they intend to convey. This provides reasonable grounds to consider them real news. However, our model identifies irregularities in their social relations, leading to their classification as fake news. Specifically, the comment section under the news post in Figure 9a exhibits a pattern of highly repetitive user interactions, with multiple comments containing identical or near-identical minimal responses (e.g., “Seems these cops think too much and know too little.”). Similarly, Figure 9b exhibits an analogous pattern, indicating that our model can effectively identify evidence of fake news from social relationships and flag news items accordingly.

Although social relations have proven instrumental in fake news detection, the intrinsic characteristics of textual and visual entities remain indispensable and should not be neglected. In Figure 9c,d, “Ottawa”, “Sydney”, and “Lindt Cafe” are key pieces of information that enable readers to recognize whether the news is true or not. Nevertheless, not all readers are familiar with the meaning of these entities. Moreover, it is not possible to determine via simple observation whether entities in the text and images convey similar messages to readers. Our model can extract entities from both images and text while performing comprehensive analysis of textual and visual features, thereby contributing to more effective fake news detection.

## 6. Conclusions

This study introduces an innovative Semantic-enhanced Cross-modal Co-attention Network (SCCN) designed for enhanced fake news detection. The proposed SCCN introduces social relations with structure information and employs a co-attention mechanism to achieve cross-modal fusion with text and images. We not only successfully enhance text and images via entity extraction but also utilize an improved GAT to extract the structural features of social relations. Moreover, we deduce hidden links in the social relation graphs and remove the possible noisy links. We utilize a perturbation mechanism to enhance the robustness of the SCCN. Furthermore, we adopt cross-entropy as our optimization objective and conduct comprehensive information-theoretic analyses of both the model architecture and experimental outcomes. The results of our experiments demonstrate that the performance of the proposed SCCN is better than that of various baselines. The source code for SCCN can be found at https://github.com/asufdahu/SCCN (accessed on 20 June 2025).

In subsequent research, we intend to introduce external knowledge to improve the representation capability of the model, which can enhance the confidence coefficient of the entities and feature vectors. In addition, we will incorporate Mutual Information Maximization (MIM) into our framework to enhance model performance by optimizing the mutual information between input and output representations.

## Figures and Tables

**Table 1:** Statistics of the datasets used in our experiments after data cleaning.

Statistics	Non Rumors	Rumors	Images	Users	Comments
PHEME	1428	590	2018	894	7388
Weibo	877	590	1467	985	4534

**Table 2:** Performance comparison between SCCN and other methods. The global best and second-best results are highlighted in bold and underlined, respectively. SCCN-F and SCCN-R represent the results of the fake and real classes in prediction performance. To validate the stability of our method, we conducted experiments under five different randomized seeds, then confirmed the statistical significance of the pairwise differences in the SCCN relative to the strongest baseline using a *t*-test *p*-value < 0.01 (1 × 10 ^−2^).

	PHEME [34]				Weibo [35]			
Method	Accuracy	Precision	Recall	F1 Score	Accuracy	Precision	Recall	F1 Score
EANN [2]	0.771	0.714	0.700	0.704	0.809	0.802	0.797	0.799
MVAE [1]	0.776	0.735	0.722	0.728	0.717	0.705	0.702	0.703
QSAN [17]	0.751	0.699	0.658	0.669	0.710	0.710	0.675	0.676
SAFE [15]	0.815	0.799	0.795	0.797	0.849	0.850	0.849	0.849
EBGCN [21]	0.830	0.813	0.793	0.798	0.831	0.854	0.818	0.814
GLAN [13]	0.833	0.812	0.771	0.785	0.824	0.824	0.809	0.813
MPFN [5]	0.833	0.827	0.821	0.810	0.838	0.865	0.878	0.882
KMAGCN [22]	0.864	0.851	0.855	0.853	0.849	0.847	0.850	0.849
MFAN [10]	0.887	0.870	0.856	0.861	0.889	0.889	0.881	0.883
SCCN-F (ours)	0.904	0.918	0.949	0.933	0.905	0.883	0.972	0.925
SCCN-R (ours)	0.904	0.865	0.796	0.829	0.905	0.949	0.802	0.869
**SCCN (ours)**	**0.904**	**0.902**	**0.904**	**0.903**	**0.905**	**0.909**	**0.905**	**0.903**
*p*-value	5.44 × 10 ^−5^	3.32 × 10 ^−5^	4.04 × 10 ^−8^	2.43 × 10 ^−7^	6.49 × 10 ^−3^	3.10 × 10 ^−4^	1.80 × 10 ^−5^	6.65 × 10 ^−3^

**Table 3:** Results showing the practical deployability of SCCN.

Dataset	Time (s/epoch)	Inference Latency (s/epoch)	Memory (GB)
PHEME [34]	30.24	0.868	1.99
Weibo [35]	29.08	0.869	2.45

**Table 4:** Performance comparison between the full SCCN model and different variants.

Method		-w/o DPS	-w/o SRG	-w/o IEM	-w/o CMF	SCCN
PHEME [34]	Accuracy	0.879	0.880	0.875	0.886	0.904
	Precision	0.877	0.881	0.878	0.884	0.902
	Recall	0.877	0.880	0.875	0.885	0.904
	F1 Score	0.878	0.881	0.876	0.882	0.903
Weibo [35]	Accuracy	0.875	0.871	0.878	0.888	0.905
	Precision	0.874	0.871	0.878	0.898	0.909
	Recall	0.875	0.871	0.877	0.888	0.905
	F1 Score	0.874	0.870	0.878	0.885	0.903

**Table 5:** Accuracy and F1 score results with different text lengths on the two datasets. SCCN-20 means that the text length is set to 20.

Dataset	Metric	SCCN-20	SCCN-30	SCCN-40	SCCN-50	SCCN-60
PHEME [34]	Accuracy	0.743	0.763	0.865	0.904	0.875
	F1 Score	0.733	0.744	0.867	0.903	0.873
Weibo [35]	Accuracy	0.607	0.800	0.874	0.905	0.817
	F1 Score	0.458	0.802	0.873	0.903	0.802

## Data Availability

The datasets can be found at https://figshare.com/articles/PHEME_dataset_of_rumours_and_non-rumours/4010619 (accessed on 24 October 2016) and https://drive.usercontent.google.com/download?id=14VQ7EWPiFeGzxp3XC2DeEHi-BEisDINn&export=download (accessed on 22 May 2021).
